# The effect of Norouz holiday on anthropometric measures and body composition

**DOI:** 10.1186/s40200-015-0134-5

**Published:** 2015-02-25

**Authors:** Moloud Payab, Shirin Hasani-Ranjbar, Hoda Zahedi, Mostafa Qorbani, Zahra Shateri, Bagher Larijani, Ahmadreza Soroush

**Affiliations:** Obesity and Eating Habits Research Center, Endocrinology and Metabolism Molecular -Cellular Sciences Institute, Tehran University of Medical Sciences, Tehran, Iran; Endocrinology and Metabolism Research Center, Endocrinology and Metabolism Clinical Sciences Institute, Tehran University of Medical Sciences, Tehran, Iran; Department of Community Medicine, Alborz University of Medical Sciences, Karaj, Iran; Non-Communicable Diseases Research Center, Endocrinology and Metabolism Population Sciences Institute, Tehran University of Medical Sciences, Tehran, Iran; EMRI (Endocrinology and Metabolism Research Institute), Shariati Hospital, 5th Floor, North Kargar Ave., Tehran, 14114 Iran

**Keywords:** Holiday, Body weight, Body composition, Obesity, Metabolic syndrome, Norouz Holiday

## Abstract

**Background:**

This study examined the effect of holiday season on the anthropometric measures in Shariati hospital staff.

**Methods:**

This study was conducted in 2014 on 66 subjects, aged 21–68 years. Weight, height, waist circumference (WC), hip circumference (HC), waist -to-hip ratio (WHR), waist-to- height ratio (WHtR) and physical activity were measured. Bioelectrical impedance analysis (BIA) method and the Tanita body composition analyzer were used to analyze body composition.

**Results:**

In this study, 80.3% of subjects were female and 19.7% were male. The mean age of subjects was 39.5 (SD: 9.7) years (range: 21–68). The percentage of overweight and obesity in the study population were 40.9% and 12.1%, respectively. After the holiday, participants gained 0.58 kg of weight and 0.19 units of BMI) P < 0.001). The average of WC, WHR and WHtR also increased after the holiday; however they were not statistically significant. Basal metabolic rate and fat free mass raised significantly, but the amount of fat mass decreased after the holiday.

**Conclusion:**

The holiday season is a critical period for weight gain and body fat. It’s highly recommended to weight daily during the holiday season and to increase their physical activity while limiting high-calorie foods.

## Background

Today, obesity is a major health concern throughout the world. The prevalence of obesity in people over 20 years of age has increased 6.4% to 12% during 1980 to 2008 [1]. The prevalence of obesity is increasing not only in developed countries but also in developing countries. Iran, like other developing countries is involved in the epidemic of obesity and its complications. In 2007, a national survey in 28 provinces showed that 28.6% of subjects were overweight and 10.8% of those were obese [2].

Usually an imbalance between energy intake and energy expenditure can contribute to weight gain over the years. This often occurs continually throughout the year and sometimes during certain periods of the time such as holidays [3]. With the rapid increase in obesity rates, determining the critical period of weight gain and effects of changes in body composition is necessary [4]. the risk of overweight and obesity is increased during the holidays [5]. Usually during this period energy intake is increased while physical activity is reduced [6]. Smith et al. assessed changes in BMI during holidays in their study and concluded that a small but significant increase in BMI occurred among children [7]. The Texas Medical Association reported that based on the evidence of numerous studies an average weight gain of 3.6 kg occurs in Americans from Thanksgiving to New Year’s Day [8]. In another study, it was shown that healthy weight subjects, have an average weight gain of 0.9 pounds during the holiday season, meanwhile this is 5 pounds in overweight subjects [9].

The media often reported that 5 pounds of body weight are gained during the holidays, but a few research is done to evaluate changes in weight and body composition [3,8]. Norouz refers to Iranian new year and it marks the start of a new solar year and the arrival of spring. This ceremony occurs in late of March and lasts for around six days and provides Iranian families with an opportunity to visit each other. So far, no study has conducted on weight changes over the Norouz holiday in Iran. Therefore, the purpose of this study was to examine the effects of the holiday season on weight and body composition.

## Method

In this study, 69 staff of the Shariati hospital were selected via convenience sampling method from a total of 87 participants visited in the 1st phase of the study. Participants were healthy and free from any disease that affects the metabolism or body weight. After a full explanation of the study objectives for subjects, written consent was obtained from them. Data collection and measurements were carried out during two visits. The first visit was carried out in mid March before the holiday and the second one was postponed until after holiday in early/mid April (The average interval of two measurements was 45 days). During the first visit, demographic data were collected (such as age, family size, and sedentary lifestyle, physical activity, occupation and education level). The physical activity level was measured by a short form international physical activity questionnaire (IPAQ) [10].

During each visit, weight, height, Body Mass Index (BMI), waist circumference (WC), hip circumference (HC), waist-to-hip ratio (WHR) and waist to height ratio (WHtR) were measured. Weight was measured with minimal clothing and without shoes with 0.1 kg accuracy. Standing height of the subjects was measured without shoes with 0.1 cm accuracy. WC and HC were measured using a plastic flexible tape on the distance between the smallest area below the rib cage and the iliac crest. WHR was calculated by dividing WC (in cm) by HC (in cm). WHtR was also obtained by dividing WC (in cm) by height (in cm). Abdominal obesity was considered as WHtR more than 0.5 [11]. BMI was calculated as weight (kg)/height2 (m2). BMI was categorized as underweight (<18.9 kg/m2), normal (between 18.9 and 24.9 kg/m2), overweight (between 25 and 29.9 kg/m2) and obese (>30 kg/m2) [11]. Bioelectrical impedance analysis (BIA) method and Tanita body composition analyzer (Model BC-418MA) were used to analyze body composition, which leads this Method to be simple and non-invasive.

Quantitative variables were indicated as mean ± SD (standard deviation) and qualitative variables were indicated as numbers and percentages and comparison of mean of quantitative variables was performed by T-test using SPSS software version 16. P-values of less than 0.05 was considered as statistically significant.

## Results

This study was conducted on 66 subjects of Shariati hospital staff (from the total of 87 participants, 66 subjects completed the two visits), 80.3% of whom were female and 19.7% were male. The average age of subjects was 39.5 (SD: 9.7) years (range: 21–68). Demographic data of the participants are summarized in Table 1. The percentage of overweight and obesity in the study population were 40.9% and 12.1%, respectively.

**Table 1:** **Demographic data of participants**

**Variables**	
**Means ± SD**	
**Age**	39.5 (9.7)
**Family size**	3.58 (1.3)
**Average of working hours**	8.71 (2.8)
**Average of sleep hours**	6.94 (1.11)
**Number (percent)**	
**Sex**	
Female	53 (80.3%)
Male	13 (19.7%)
**Marital status**	
Single	15 (22.7%)
Married	47 (71.2%)
Divorced and widow	4 (6%)
**Occupation Status**	
Worker	13 (19.7%)
Secretary	10 (15.2%)
Nurse	11 (16.7%)
Researcher	12 (18.2%)
Government employee	18 (27.3%)
MD	2 (3%)
**Level of education**	
School	7 (10.6%)
Diploma	23 (34.8%)
Bachelor	22 (33.3%)
Master and MD	14 (21.2%)
**Physical activity**	
Mild	29 (43.9%)
Moderate	32 (48.5%)
Severe	4 (6.1%)

The average interval of two measurements was 45 days. During this time, a small but significant increase of 0.58 kg in weight and 0.19 units of BMI was reported during this time. The average of WC, WHR and WHtR also increased after the holidays, but these increases were trivial. The changes in anthropometric measures between-before- and after the holiday are shown in Table 2. Basal metabolic rate and fat free mass increased significantly, but the amount of fat mass decreased after the holiday.

**Table 2:** **Anthropometric measures and body composition changes**

**Variables**	**Before**	**After**	**Differences**	**P-value**
	**Mean ± SD**	**Mean ± SD**		
Weight (kg)	67.42 ± 12.33	68 ± 12.22	+0.58	<0.001*
Waist Circumference (cm)	85.48 ± 10.58	86.1 ± 11.07	+0.62	0.194
Hip Circumference (cm)	101.32 ± 7.16	101.22 ± 6.89	−0.1	0.685
BMI	25.81 ± 4.53	26.0 ± 4.48	+0.19	<0.001*
Waist to Hip Ratio (cm)	0.842 ± 0.07	0.85 ± 0.08	+0.008	0.123
Waist to Height Ratio (cm)	0.528 ± 0.064	0.532 ± 0.067	+0.004	0.218
Basal Metabolic Rate	1499.19 ± 659.92	1518.53 ± 666.29	+19.34	<0.001*
Percent of Fat (%)	30 ± 7.84	29.5 ± 7.84	−0.5	0.008*
Fat Mass (kg)	20.54 ± 8.26	20.36 ± 8.08	−0.18	0.230
Fat Free Mass (kg)	47.05 ± 8.58	47.78 ± 8.8	+0.73	<0.001*

*P ≤ 0.05 is considered as significant.

## Discussion

Although a weight gain is generally expected during the holiday season. Very few scientific studies have been done so far on this subject. This study examined the holiday season on the anthropometric measures in Shariati hospital staff for the first time in Iran.

In the current study, we found that, on average, 0.58 kg weight was gained. This is consistent with other studies; in which a weight gain of less than 0.5 kg during this period was reported [3,8,12,13]. Only in the study of Reid and Hackett a 0.93 kg weight gain was reported in subjects [14]. However, in another study conducted on students in University of Oklahoma-Norman campus; no significant weight gain was observed [4]. One more study showed that in overweight and obese people, weight gain is more significant than those with normal weight [4,15]. This study also found that subjects who have had weight gain, usually may return to their previous weight within a short time, but over time, their body fat increases without changing in BMI [4,15]. Racette et al. measured body weight in 48 subjects in all year and found that the subjects gained weight averaged 3 g/d, but in weekend gained weight 60 g/d [16]. That is a similar situation during the holiday season.

More studies have usually only measured body weight, however in Hull et al. and Wagner et al. studies body composition was also considered [4,15]. The results showed a small decrease in the percentage of body fat decrease which is probably due to the small sample size. In another study, fat mass and percent of fat mass increased after the holidays. There is also a positive association between body weight and fat mass, which coincided with increased body weight, body fat increases [4]. Wagner et al. observed no significant differences in percent of body fat before and after 6 weeks [15].

In this study, WC, WHR, WHtR increased trivially. In the other study on subjects a significant, though small, increase in waist circumference was observed [15].

### Strengths and limitations

Considering the fact that the current study was quite unique in Iran, one of the strengths of this study was that participants were unaware of the purpose of the study; the other strong point was measuring body composition in subjects.

Meanwhile, the limitations of this study included small sample size.

## Conclusion

The holiday season is a period for weight gain and body fat. This study and other studies showed that body weight increases after the holidays, but a return to the baseline weight may happen, while the amount of body fat, which can lead to other diseases, may increase over time. It’s highly recommended that weight is measured during the Norouz holidays and to physical activity is raised while high calorie foods are avoided.

## Abbreviations

WC: Waist circumference
HC: Hip circumference
WHR: Waist -to-hip ratio
WHtR: Waist-to- height ratio
BIA: Bioelectrical impedance analysis
BMI: Body Mass Index
IPAQ: International physical activity questionnaire
